# Sex Differences in the Clinical Features, Risk Factors, and Outcomes of Intracerebral Hemorrhage: a Large Hospital-based Stroke Registry in China

**DOI:** 10.1038/s41598-017-00383-6

**Published:** 2017-03-21

**Authors:** Yonghong Xing, Zhongping An, Xianghui Zhang, Ning Yu, Wenjuan Zhao, Xianjia Ning, Jinghua Wang

**Affiliations:** 1 Department of Neurology, Tianjin Huanhu Hospital, Tianjin, China; 2 Tianjin Key Laboratory of Cerebral Vascular and Neurodegenerative Disease, Tianjin, China; 3 Department of Epidemiology, Tianjin Neurological Institute & Department of Neurology, Tianjin Medical University General Hospital, Tianjin, China

## Abstract

Intracerebral hemorrhage (ICH) is common in China. However, the sex differences in clinical features, risk factors, and outcomes of ICH remain controversial. Between 2005 and 2014, we recruited patients with primary ICH in Tianjin, China, and evaluated sex differences in clinical features, risk factors, and outcomes at 3, 12, and 36 months after ICH. The 1,325 patients included 897 men (67.7%) and 428 women (32.3%). The mean age at ICH onset was younger among men (59.14 years) than among women (63.12 years, P < 0.001). Men were more likely to have a hematoma in the basal ganglia, while women were more likely to have one in the thalamus. Women had higher frequencies of urinary tract infections, diabetes mellitus, cardiovascular diseases, and obesity. Men had a greater risk of death at 3 months after ICH. However, no sex differences were observed for mortality at 12 and 36 months after ICH or for recurrence and dependency at 3, 12, and 36 months after ICH. These findings suggested that it crucial to strengthen management of AF and complications in patients with ICH, especially management of blood pressure in men for reducing the mortality rates and the burden of ICH in China.

## Introduction

Several studies have evaluated sex-related differences in functional outcomes among patients with intracerebral hemorrhage (ICH)^1–3^; however, mortality rates and outcomes following ICH remain controversial. For example, some studies have reported a higher mortality rate among women^4–6^, while others have reported a higher mortality rate among men^7, 8^. Other studies have reported no sex-related differences in mortality after ICH^3, 9, 10^. Moreover, there are limited data regarding sex-related differences in long-term outcomes (particularly outcomes at >1 year), including recurrence and dependency rates after ICH. Therefore, the present study aimed to evaluate sex-related differences in functional outcomes (mortality, dependency, and recurrence rates) in the short-term (3 months), medium-term (12 months), and long-term (36 months) after ICH.

## Results

**Figure 1:**
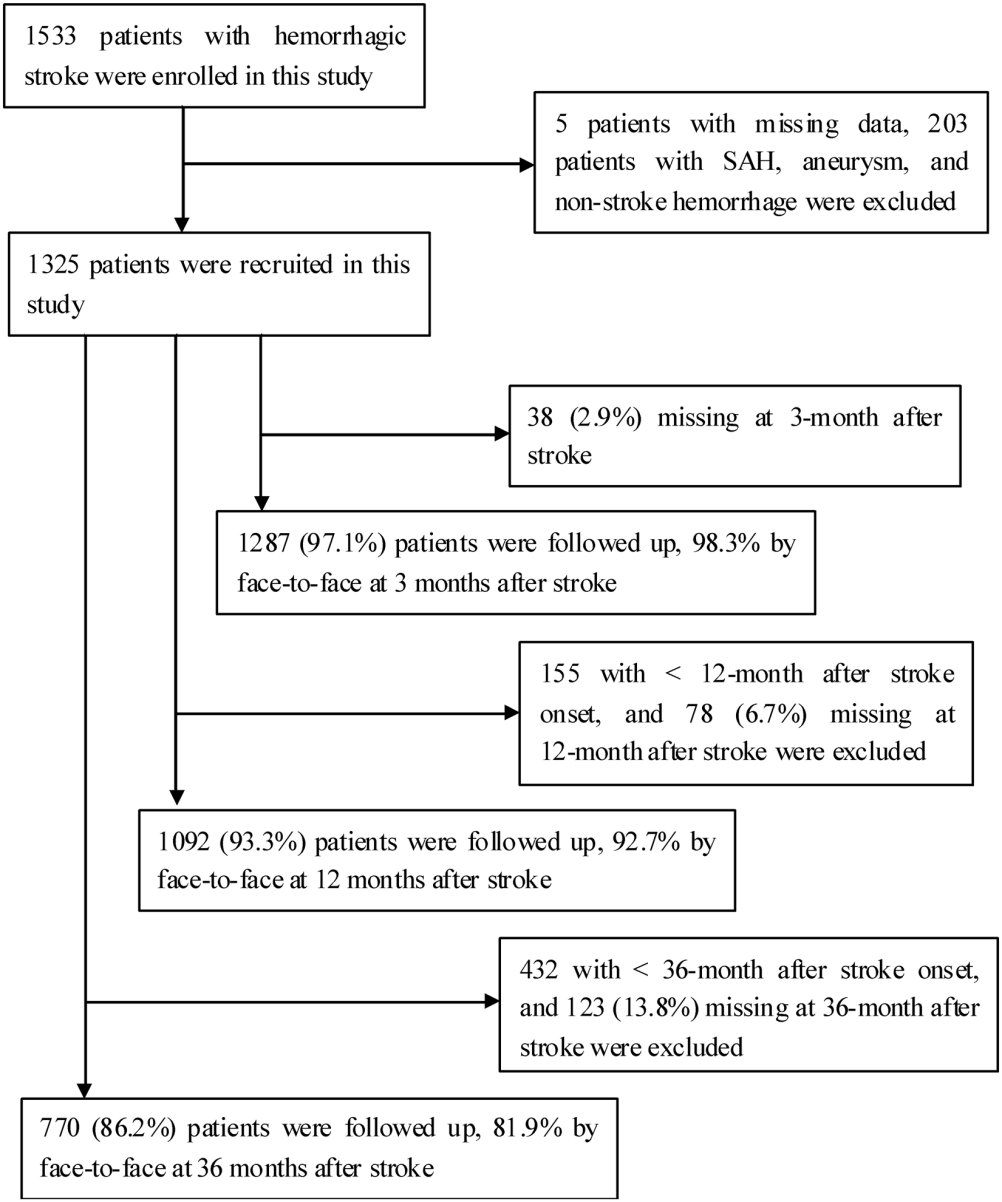
Response rates were 97.1% at 3 months after ICH, 92.9% at 12 months after ICH, and 86.2% at 36 months after ICH, respectively. ICH, intracerebral hemorrhage.

During the study period, 1,533 consecutive patients diagnosed with first-ever hemorrhagic stroke were registered in our database. Among these patients, 1,330 patients fulfilled our inclusion criteria, and we analyzed the records of 1,325 patients with complete data. The patient selection flow chart is shown in Fig. 1. Of the 1,325 patients who had experienced at least 3 months after stroke onset, 1,287 patients (97.1%) completed the 3-month follow-up; among 1,170 patients who had experienced at least 12 months after stroke onset, 1,092 patients (93.3%) completed the 12-month follow-up; and among 893 patients who had experienced at least 36 months after stroke onset, 770 patients (86.2%) completed the 36-month follow-up.

The present study included 897 men (67.7%) and 428 women (32.3%). The mean age at ICH onset was younger in men than in women (59.14 years vs. 63.12 years, respectively), and men were more likely to be <45 years of age at ICH onset (11.5% vs. 3.0%; P < 0.001 for all). Men were also more likely to have a basal ganglia hematoma (47.4% vs. 36.2%; P < 0.001), although women were more likely to have a thalamus hematoma (21.7% vs. 12.4%; P < 0.001). Moreover, the frequency of number for multi-hematoma was similar between men and women (8.7% vs. 11.7%, P = 0.085). Regarding in-hospital complications, urinary tract infections were more prevalent in women than in men (3.7% vs. 1.6%; P = 0.013), although there were no other statistically significant sex-related differences in complication rates. Women had significantly greater neurological function deficits, with lower Barthel indices (BIs) and higher modified Rankin scale (mRS) scores at admission; men and women had similar National Institutes of Health Stroke Scale (NIHSS) scores.

There were higher prevalence rates of diabetes mellitus (DM) (22.9% vs. 17.7%; P < 0.001), cardiovascular disease (26.6% vs. 17.7%; P < 0.001), and obesity (15.7% vs. 10.8%; P = 0.012) in women than in men; however, there were no other significant sex-related differences in medical history factors (P > 0.05 for all). The rates of current smoking status (47.2% vs. 11.9%; P < 0.001) and alcohol consumption (30.5% vs. 1.6%; P < 0.001) were higher in men than in women. Women had significantly higher levels of total cholesterol (TC), low-density lipoprotein cholesterol (LDL-C), fasting glucose (FG), and glycosylated hemoglobin (HbA1c) (P < 0.05 for all), and men had significantly higher diastolic blood pressure (DBP). We did not observe any significant sex-related differences in levels of triglycerides (TG), high-density lipoprotein cholesterol (HDL-C), or systolic blood pressure (SBP) (Table 1).

**Table 1:** Sex differences in demographical and clinical characteristics.

Characteristics	Men	Women	P
Numbers, n(%)	897 (67.7)	428 (32.3)	
Age, year, mean(SD)	59.14 (12.74)	63.12 (11.62)	<0.001
Age group, n(%)			<0.001
<45 years	103 (11.5)	13 (3.0)	
45–59 years	393 (43.8)	163 (38.1)	
≥60 years	401 (44.7)	252 (58.9)	
Hematoma location, n(%)			<0.001
Basal ganglia	425 (47.4)	155 (36.2)	
Lobar	98 (10.9)	47 (11.0)	
Thalamus	111 (12.4)	93 (21.7)	
Brainstem	142 (15.8)	62 (14.5)	
Cerebellum	43 (4.8)	21 (4.9)	
≥2 locations	78 (8.7)	50 (11.7)	
Number of the hematoma, n(%)			0.085
Single hematoma	819 (91.3)	378 (88.3)	
Multi- hematoma	78 (8.7)	50 (11.7)	
Stroke severity, n(%)			0.219
Mild	401 (44.7)	176 (41.1)	
Moderate/Severe	496 (55.3)	252 (58.9)	
Neurological function deficit			
NIHSS	9.00 (0–42)	9.00 (0–40)	0.265
BI	40.00 (0–100)	30.00 (0–100)	0.001
mRS	4.00 (0–6)	4.00 (1–6)	0.009
Complication in hospital, n(%)	188 (21.0)	94 (22.0)	0.676
Pulmonary infection	131 (14.6)	61 (14.3)	0.865
Urinary Infection	14 (1.6)	16 (3.7)	0.013
GI hemorrhage	65 (7.2)	29 (6.8)	0.755
Seizure	4 (0.4)	2 (0.5)	0.957
Electrolyte disturbance	30 (3.3)	15 (3.5)	0.880
Liver/renal toxicity	16 (1.8)	3 (0.7)	0.121
Medical history, n(%)			
Hypertension	766 (85.4)	370 (86.4)	0.608
Diabetes	159 (17.7)	98 (22.9)	0.026
Atrial fibrillation	23 (2.6)	16 (3.7)	0.237
Cardiovascular disease	159 (17.7)	114 (26.6)	<0.001
Obesity	97 (10.8)	67 (15.7)	0.012
Current smoking	423 (47.2)	51 (11.9)	<0.001
Alcohol consumption	274 (30.5)	7 (1.6)	<0.001
Blood pressure, mmHg, mean(SD)			
SBP	165.27 (27.08)	165.49 (26.69)	0.926
DBP	93.75 (15.05)	87.89 (13.24)	<0.001
Laboratory, mmol/l, mean(SD)			
Total cholesterol	4.94 (1.04)	5.37 (1.07)	<0.001
Triglyceride	1.60 (1.20)	1.47 (1.02)	0.068
High density lipoprotein cholesterol	1.22 (0.36)	1.35 (0.36)	<0.001
Low density lipoprotein cholesterol	2.93 (0.85)	3.15 (0.86)	<0.001
Fasting glucose	6.37 (2.39)	6.94 (2.90)	0.002
Glycosylated hemoglobin	6.10 (1.17)	6.41 (1.32)	0.017

In the univariate analysis, we observed significant sex-related differences in mortality rates at each follow-up period. The mortality rates at 3 (13.5% vs. 9.0%, P = 0.021), 12 (17.2% vs. 13.4%, P = 0.111), and 36 months (25.3% vs. 21.9%, P = 0.307) were higher for men than for women. The unadjusted OR (95%CI) was 1.58 (1.07–2.33) at 3 months after ICH. However, there were no significant sex-related differences in recurrence or dependency rates (Table 2).

**Table 2:** Sex differences in outcomes at 3, 12, 36 months after ICH.

Outcomes	Men	Women	Unadjusted OR (95% CI)	P
3 months (n = 1287)				
Mortality	118 (13.5)	37 (9.0)	1.58 (1.07, 2.33)	0.021
<45 years	11 (10.9)	2 (15.4)	0.67 (0.13, 3.44)	0.631
45–59 years	41 (10.6)	8 (5.0)	2.27 (1.04, 4.96)	0.036
≥60 years	66 (17.0)	27 (11.3)	1.60 (0.99, 2.59)	0.053
Recurrence	27 (2.1)	7 (1.7)	1.84 (0.97, 4.27)	0.147
<45 years	0	0	—	—
45–59 years	16 (4.1)	1 (0.6)	6.88 (0.90, 52.29)	0.063
≥60 years	11 (2.9)	6 (2.5)	1.14 (0.42, 3.12)	0.801
Dependency	271 (35.7)	150 (40.0)	0.83 (0.65, 1.07)	0.159
<45 years	20 (22.2)	6 (54.5)	0.24 (0.06, 0.88)	0.021
45–59 years	121 (35.0)	41 (26.8)	1.47 (0.97, 2.24)	0.072
≥60 years	130 (40.2)	103 (48.8)	0.71 (0.50, 1.00)	0.051
12 months (n = 1092)				
Mortality	129 (17.2)	46 (13.4)	1.34 (0.93, 1.93)	0.111
<45 years	11 (13.4)	2 (15.4)	0.70 (0.13, 3.66)	0.669
45–59 years	41 (12.5)	11 (8.8)	1.49 (0.74, 2.99)	0.265
≥60 years	77 (22.6)	33 (15.9)	1.54 (0.92, 2.42)	0.058
Recurrence	74 (9.9)	27 (7.9)	1.28 (0.81, 2.03)	0.288
<45 years	2 (2.4)	1 (9.1)	0.25 (0.02, 3.01)	0.241
45–59 years	33 (10.1)	10 (8.0)	1.29 (0.62, 2.70)	0.498
≥60 years	39 (11.5)	16 (7.7)	1.55 (0.84, 2.85)	0.158
Dependency	172 (27.7)	84 (28.0)	0.98 (0.72, 1.34)	0.923
<45 years	6 (8.5)	5 (55.6)	0.07 (0.02, 0.35)	<0.001
45–59 years	78 (27.3)	25 (21.6)	1.37 (0.82, 2.28)	0.234
≥60 years	88 (33.3)	54 (30.9)	1.12 (0.74, 1.69)	0.587
36 months (n = 770)				
Mortality	136 (25.3)	51 (21.9)	1.21 (0.84, 1.75)	0.307
<45 years	11 (18.6)	2 (28.6)	1.50 (0.15, 2.99)	0.532
45–59 years	44 (19.3)	12 (13.8)	0.57 (0.10, 3.35)	0.253
≥60 years	81 (32.4)	37 (26.6)	1.32 (0.83, 2.09)	0.235
Recurrence	159 (29.6)	62 (26.6)	1.16 (0.82, 1.64)	0.398
<45 years	7 (11.9)	2 (28.6)	0.34 (0.06, 2.08)	0.223
45–59 years	61 (26.8)	19 (21.8)	1.31 (0.73, 2.35)	0.370
≥60 years	91 (36.4)	41 (29.5)	1.37 (0.88, 2.14)	0.168
Dependency	205 (50.9)	87 (47.5)	1.14 (0.81, 1.62)	0.455
<45 years	19 (39.6)	3 (60.0)	0.44 (0.07, 2.86)	0.378
45–59 years	87 (47.0)	31 (41.3)	1.26 (0.73, 2.17)	0.403
≥60 years	99 (58.2)	53 (51.5)	1.32 (0.80, 2.15)	0.274

Furthermore, the results stratified by age groups showed that mortality at 3 months after ICH was significantly higher in men than in women among those aged 45–59 years, and the dependency rate was greater in men than in women at 3 and 12 months among patients aged <45 years (Table 2).

Table 3 presents the adjusted ORs for men at the 3-, 12-, and 36-month follow-ups. Men had a higher risk of death at 3 months after ICH (OR, 2.32; 95% confidence interval [CI], 1.45–3.72; P < 0.001) than did women after adjustment for those covariates found to be significant in the univariate analysis, including age, stroke severity, hematoma number, hypertension, atrial fibrillation (AF), dyslipidemia, complications, current smoking, and alcohol consumption. Moreover, severe stroke, AF, and complications were independent risk factors for mortality at 3 months after ICH. However, there were no significant sex differences for recurrence and dependency rates at 3, 12, and 36 months after ICH. Severity, AF, complications, and multiple hematomas were associated with high risk of mortality at 12 months after ICH; severity, complications, and multiple hematomas were associated with a high risk of mortality at 36 months after ICH. Furthermore, older age, greater severity, and complications were determinants of dependency at 3 and 12 months after ICH, but severity and AF were determinants of dependency at 36 months after ICH.

**Table 3:** Adjusted OR (95% CI) for associated factors of outcomes at 3, 12, and 36 months after stroke.

Risk Factors	References	Mortality	Recurrence	Dependency
*3 months*				
Men	Women	2.32 (1.45, 3.72)		—
Age group	<45 years			
45–59 years		0.84 (0.39, 1.78)		1.15 (0.90, 3.67)
≥60 years		1.33 (0.63, 2.78)		2.67 (1.54, 4.62)
Severity	Mild			
Moderate		3.12 (1.51, 6.48)		7.57 (5.50, 10.42)
Severe		35.12 (17.85, 69.09)		18.71 (11.84, 29.56)
Hypertension	No	1.34 (0.80, 1.60)		1.22 (0.78, 1.90)
AF	No	2.64 (1.01, 6.89)		—
Dyslipidemia	No	—		0.82 (0.57, 1.18)
Complication	No	2.19 (1.44, 3.35)		1.70 (1.17, 2.48)
Multi-hematoma	Single	1.36 (0.76, 2.45)		1.44 (0.88, 2.36)
Alcohol consumption	Never	—		0.88 (0.63, 1.25)
*12 months*				
Age group	<45 years			
45–59 years		0.76 (035, 1.65)		2.36 (1.18, 4.74)
≥60 years		1.26 (0.59, 2.68)		3.18 (1.59, 6.35)
Severity	Mild			
Moderate		2.69 (1.43, 5.03)		3.61 (2.57, 5.09)
Severe		25.95 (14.37, 46.89)		5.26 (3.32, 8.34)
Hypertension	No	0.39 (0.22, 0.67)		—
AF	No	3.03 (1.11, 8.23)		—
Dyslipidemia	No	—		—
Complication	No	2.47 (1.63, 3.76)		1.50 (1.02, 2.20)
Multi-hematoma	Single	1.89 (1.04, 3.44)		0.97 (0.55, 1.73)
Current smoking	Never	0.96 (0.59, 1.56)		—
Alcohol consumption	Never	0.80 (0.43, 1.50)		—
*36 months*				
Age group	<45 years			
45–59 years		0.87 (0.40, 1.92)	2.16 (1.00, 4.55)	1.21 (0.66, 2.22)
≥60 years		1.89 (0.87, 4.08)	3.25 (1.56, 6.78)	1.76 (0.96, 3.23)
Severity	Mild			
Moderate		1.81 (1.06, 3.10)		1.45 (1.01, 2.09)
Severe		14.10 (8.41, 23.64)		1.93 (1.13, 3.28)
AF	No	2.34 (0.83, 6.56)		9.90 (1.25, 18.46)
Complication	No	2.07 (1.36, 3.15)		1.08 (0.70, 1.65)
Multi-hematoma	Single	2.61 (1.45, 4.71)		—
Alcohol consumption	Never	0.86 (0.49, 1.51)		—

## Discussion

To our knowledge, this is the first study to examine sex-related differences in long-term outcomes after ICH. Using a large hospital-based registry, we evaluated mortality, recurrence, and dependency rates at 3, 12, and 36 months after ICH. Our findings revealed various sex-related differences in patients’ demographic and clinical characteristics. Women were more likely than men to be older; have a greater frequency of urinary tract infections, DM, cardiovascular disease, obesity; and have higher levels of TC, HDL-C, LDL-C, FG, and HbA1c. However, men were more likely than women to be younger and have higher DBP. Men aged 45–59 years had significantly higher mortality at 3 months after ICH; male sex was an independent risk factor for mortality after adjusting for covariates. Moreover, severity and complications were determinants of mortality and dependency after ICH. Recurrence was associated with older age only at 36 months after ICH.

Over the past few decades, the incidence of ICH in developed countries has remained unchanged or decreased^2, 11–14^. However, the trends in China are inconsistent, as the incidence of ICH has decreased in urban areas but increased in rural areas (for both sexes)^15, 16^. Interestingly, studies have also found that men are more likely to experience their first stroke at a younger age than women are. A study with exclusion criteria similar to those utilized in the present study reported that women were on average 8 years older than men at ICH onset^11^. Another study found that women were on average 6 years older than men at the time of stroke^17^.

Similarly, we found that men were approximately 4 years younger at ICH onset than women were. However, conflicting trends have been reported in other studies; an American study reported that women in North Carolina experienced ICH 4 years before men did^18^, and other studies have reported no significant differences between sexes in the age of onset^19, 20^. In the present study, men aged 45–59 years had a significantly higher mortality at 3 months after ICH. It is possible that the neuroprotective effects of female gonadal hormones delay the onset of ICH among women, as these hormones play roles in decreasing lipid levels and altering rapid vasomotor responses in vessel walls^21–23^. There is a lower rate of hormone replacement therapies in Chinese women; the rate of regularly using hormone replacement therapies (1 year and over) was 1.1% in mainland China^24^ and 13.5% in Taiwan^25^ among postmenopausal women. In this study, none of the women were taking hormone replacement therapies; therefore, the neuroprotective effects of estrogen may play an important role in delaying the presence of ICH among women.

Previous studies have demonstrated that stroke burden is higher among women than among men due to higher rates of pre- and post-stroke disability^17, 18, 26, 27^. However, sex-related differences in stroke outcomes may be due to the study design, which include setting (population-based or hospital-based), population (Asian or Western), inclusion criteria, analytical methods, and duration of follow-up^19^. For example, some studies have reported higher mortality rates among women after ICH^4–6^, some have reported similar mortality rates between men and women after ICH^3, 9, 10^, and others have reported higher mortality rates among men after ICH^7, 8^. In the present study, we observed higher mortality rates among men at 3 months after ICH. This higher mortality rate may be explained by the higher prevalence of smoking and alcohol consumption and the higher DBP levels among men; in addition, lower levels of TC and HDL-C, which have been reported to be associated with a higher risk of death after ICH, may contribute to the higher mortality rate in men after ICH^28, 29^.

Several studies have reported that hypercholesterolemia is a protective factor for ICH. For example, the Ludwigshafen Stroke Study in Germany found that the absence of hypercholesterolemia before ICH was associated with a 22% higher mortality rate after ICH^30^. Another study identified an association between a history of hypercholesterolemia and a decreased risk of ICH^31^, and TC has been reported to be negatively associated with hemorrhagic and total stroke mortality^32^.

Recently, two studies reported the long-term mortality rates and functional outcomes among stroke patients^31, 32^. A study from the Swedish Stroke Register indicated there were higher mortality rates among women than among men at 3 and 12 months after stroke, and elderly women (aged 75 years and over) were most susceptible to deterioration, with dependency rates increasing from 23.2% to 45.5%^33^. Another Collaborative Evaluation of Rehabilitation in Stroke Across Europe Study showed that functional and motor outcomes at 5 years were equal to those 2 months after stroke. Increasing age and increasing stroke severity negatively affected outcomes^34^. However, only a few studies have reported sex-related differences in long-term (>1 year) functional outcomes after ICH. For example, one Chinese study found that women had a higher risk of dependency at 3 and 6 months after ICH^19^. In contrast, we found no sex-related differences in recurrence and dependency rates at 3, 12, and 36 months after ICH.

Although this study included a large sample of patients diagnosed with ICH and evaluated long-term outcomes, there are also several limitations. First, all patients were from a single hospital in northern China, and it cannot be assumed that our findings are representative of the general Chinese population. However, given the high incidence of ICH in northern China, our large sample provides reliable information regarding local sex-related differences in ICH outcomes. Second, we did not collect information regarding pre-stroke medications. This omission may have confounded our analysis of sex-related differences for various factors. Third, our registry did not contain information regarding hematoma volume, which could affect outcomes after ICH. However, we replaced hematoma volume with the number of hematomas, which could roughly estimate the hematoma volume. Finally, information regarding rehabilitation therapy after the acute phase was not provided in this study, but it might have an effect on the evaluation of prognosis after ICH. Finally, a follow-up rate of <90% at 36 months after ICH could impact the evaluation of outcomes at 36 months after ICH.

## Conclusions

This is the first study to evaluate sex-related differences in the clinical features, risk factors, and short- to long-term outcomes among Chinese patients diagnosed with ICH. Our findings revealed that ICH onset occurred approximately 4 years earlier in men than in women. Women were more likely than men to be older; have a greater frequency of urinary tract infections, DM, cardiovascular disease, obesity; and have higher levels of TC, HDL-C, LDL-C, FG, and HbA1c. However, men were more likely than women to be younger and have higher DBP. Men aged 45–59 years had significantly higher mortality at 3 months after ICH; male sex was an independent risk factor for mortality after adjusting for covariates. Moreover, severity and complications were determinants of mortality and dependency after ICH. Recurrence was associated with older age only at 36 months after ICH. These findings suggest that it is crucial to strengthen the management of AF and complications in patients with ICH, especially the management of blood pressure in men, to reduce mortality rates and the burden of ICH in China.

## Materials and Methods

This study evaluated data from a prospectively maintained database of patients diagnosed with ICH who were admitted to the stroke unit of Tianjin Huanhu Hospital, China, between January 2005 and September 2014. We assessed the outcomes at 3, 12 and 36 months after ICH in December 31, 2014. Of these, those patients who registered before September 30, 2014 were qualified to assess the outcomes at 3 months after ICH; those patients who registered before December 31, 2013 were qualified to assess the outcomes at 12 months after ICH; and those patients who registered before December 31, 2011 were qualified to assess the outcomes at 36 months after ICH.

A diagnosis of ICH was made according to the World Health Organization's criteria, and all diagnoses were confirmed using brain computed tomography findings^35^. All patients with ICH were admitted to the hospital within 72 h of stroke onset and were ≥18 years of age at the time of database inclusion.

We excluded patients diagnosed with subarachnoid hemorrhage, traumatic hemorrhage, and brain hemorrhage caused by vascular malformations, as well as cases of coagulopathy, aneurysmal rupture, and recurrent ICH. Furthermore, patients with premorbid dependency (defined as mRS score >2) and those who died after completing the neuroimaging diagnosis but before admission to the stroke unit were excluded from this study. For the included patients, we collected data regarding their baseline characteristics (including demographic information), clinical features, medical history, risk factors, routine laboratory test results, and outcomes 3, 12, and 36 months after ICH. Patients with ICH were treated with medications that included diuretics (mannitol, glycerol fructose, furosemide, torsemide, and albumin), antihypertensives, and medications to treat complications occurring during hospitalization.

All investigative protocols were approved by the ethics committee of Tianjin Huanhu Hospital. The procedures were performed according to approved guidelines, and a written informed consent was obtained from each patient.

The clinical features of ICH included in this analysis were hematoma location, neurological function deficits, severity, and in-hospital complications. Hematoma location was categorized as: basal ganglia, lobar, thalamus, brain stem, cerebellum, or ≥2 locations, as determined using brain computed tomography findings. Neurological function deficits were evaluated using the NIHSS score, BI, and mRS score at admission. Stroke severity was categorized into 3 groups using the NIHSS score: mild (NIHSS score ≤ 7), moderate (NIHSS score 8–16), or severe (NIHSS score ≥ 17)^36^. We also identified cases that experienced pulmonary infection, urinary tract infection, gastrointestinal hemorrhage, seizure, electrolyte disturbance, and liver/renal toxicity in the hospital. Furthermore, we collected data on patients’ levels of TC, TG, HDL-C, LDL-C, FG, and HbA1c at admission.

Regarding patient medical history, we collected data on the presence of hypertension (defined as a history of hypertension or antihypertensive drug use), DM (defined as a history of DM or hypoglycemic drug use), atrial fibrillation (AF, defined as a history of AF confirmed by at least one electrocardiogram, or the presence of arrhythmia during hospitalization), and cardiovascular disease (including coronary heart disease or myocardial infarction). We also evaluated patients’ modifiable lifestyle factors, which included current smoking status (≥1 cigarette per day for ≥1 year), alcohol consumption (≥1 drink per week for 1 year), and obesity (body mass index ≥ 30 kg/m^2^).

Patient outcomes included mortality, recurrence, and dependency rates at 3, 12, and 36 months after ICH. All outcome data were collected via in-person examinations or telephone follow-ups. Death was defined as all-cause mortality during the corresponding periods after ICH. Recurrence was defined as a new-onset vascular event, which included ICH, ischemic stroke, myocardial infarction, and venous thrombosis within 30 days after stroke. We included patients who died as a result of these vascular events, although we excluded patients with a confirmed non-vascular cause of death. Dependency was defined as an mRS score ≥3 at the time of follow-up; patients who died were excluded from the analysis of dependency rates^37^.

Follow-ups were performed according to a predetermined procedure, with trained neurologists re-examining the patients in the outpatient department at 3, 12, and 36 months after ICH. All patients completed follow-up with face-to-face interviews or with telephone interviews for patients who could not attend an in-person follow-up.

Descriptive statistics were used to evaluate sex-related differences. Age and levels of TG, TC, HDL-C, LDL-C, FG, and HbA1c are reported as means ± standard deviations, while NIHSS scores, BI, and mRS scores are reported as medians (ranges). Continuous variables were compared using the Student *t*-test or Mann-Whitney *U* test as appropriate. Dichotomous variables, including stroke subtypes, stroke severity, medical history, stroke risk factors, and outcomes at 3, 12, and 36 months after ICH, are reported as numbers (percentages). The chi-squared test was used to compare dichotomous variables. All patients missing from each follow-up period were excluded from calculations of mortality, dependency, and recurrence rates. We also excluded patients from the dependency rate calculation who completed follow-up via telephone. Sex-related differences in outcomes were assessed using logistic regression models, and the risk is reported as unadjusted ORs with 95% CIs.

A multivariate analysis of sex differences in outcomes was performed with a logistic regression model that was adjusted by those variables found to be significantly associated with outcomes at 3, 12, and 36 months after stroke in the univariate analysis, such as age, stroke severity, hematoma location, medical history, risk factors, and in-hospital complications (i.e., pulmonary infection and gastrointestinal hemorrhage). The results of the multivariate analysis are presented as adjusted ORs and 95% CIs. All statistical analyses were performed using SPSS software (version 15.0; SPSS Inc., Chicago, IL), and all tests were two-tailed. Statistical significance was defined as a P-value of <0.05.
